# Comparative assessment of medicinal plant, probiotic, and antibiotic supplementation on the growth performance, carcass traits, and meat quality of broiler chicken

**DOI:** 10.5455/javar.2026.m1052

**Published:** 2026-06-22

**Authors:** Momota Rani Debi, Shahnur Rahman Sohag, Piash Sikder, Md Abu Rayhan, Bapon Dey, Mohammad Al-Mamun, Md. Abul Hashem, Khan Md. Shaiful Islam

**Affiliations:** 1 Department of Animal Nutrition, Bangladesh Agricultural University, Mymensingh 2202, Bangladesh; 2 Department of Poultry Science, Bangladesh Agricultural University, Mymensingh 2202, Bangladesh; 3 Department of Animal Science, Bangladesh Agricultural University, Mymensingh 2202, Bangladesh

**Keywords:** Antibiotic, Broiler, Growth, Hand-mixed diet, Moringa, Yeast

## Abstract

**Objectives:** Due to rising concerns about antimicrobial resistance to antibiotic growth promoters, the use of natural feed additives is a safer alternative in poultry production. Therefore, this study aimed to examine the impact of dietary supplementation of moringa, yeast, and antibiotics on the overall productivity of broiler chicken.

**Materials and Methods:** A total of 240-day-old chicks were allocated into 4 dietary treatments (60 chicks/treatment), namely CD = Control diet, CDM = Control diet + 2.5% moringa leaf powder, CDY = Control diet + 2.5% yeast, and CDA = Control diet + 0.015% antibiotic, each having six replications (10 chicks/replication) for a period of 35 days.

**Results:** The final body weight (1736 gm) was significantly (*p* < 0.05) higher in the CDA group than in the others. In feed intake, there were no significant (*p* > 0.05) differences, but FCR and digestibility were improved in the CDA and CDY groups. Bone mineralization increased in the CDM group. Treatment groups showed significantly higher dressing yield and meat yield than the control (*p* < 0.05). The cooking loss, drip loss, water-holding capacity, TBARS value, and redness of color showed better results (*p* < 0.05) in the moringa and yeast groups than the control and antibiotic groups. The meat protein content was significantly (*p* < 0.05) higher in the CDM (21.68%) and CDY (21.10%) groups compared to CDA (20.33%). Lower fat content was recorded in the CDM (1.12%) and higher in the CDA (1.67%) group.

**Conclusions:** It can be concluded that the moringa or yeast-supplemented feeds are suitable options and appear to be promising choices for producing safe meat with improved nutritional quality.

## 1. Introduction

Poultry production is important because it is a cheap source of animal protein for humans. To enhance the production performance of poultry, antibiotic growth promoters (AGPs) have been used as feed additives that improve nutrient utilization and control subclinical diseases [1]. But the prolonged use of AGP causes resistance in pathogenic microorganisms and reduces the effects of antibiotics in the treatment of certain diseases [2]. The increase of antimicrobial-resistant bacteria in medicine and agriculture has become a significant global issue. As a result, both public and scientific concerns regarding the use of AGPs in poultry feed are increasing [3]. The European Union (EU) banned AGPs in 2006, and a similar demand for antibiotic-free meat is also growing in Bangladesh [4]. This trend has prompted the search for suitable alternatives to AGPs in poultry feed.

Several strategies have been proposed to reduce or eliminate AGPs in animal feed. However, the greatest challenge lies in identifying suitable alternatives that act through comparable mechanisms, thereby promoting growth and enhancing feed conversion efficiency. Recently, various options, including prebiotics, probiotics, herbal plants, essential oils, symbiotics, enzymes, phytobiotics, and organic acids, have been extensively evaluated as potential replacements for conventional growth promoters [5].

Probiotics are added to an animal’s diet as live microbial feed enhancements to advance intestinal microbial communities [6]. It appears to be a viable alternative to AGP, which has been used on poultry and animals to increase overall performance [7, 8]. According to Khan et al. [5], the addition of probiotics improved broiler immune response, FCR, and growth performance. Probiotics have also been shown to improve microbial balance, produce vitamins, release bacteriocins, increase feed consumption in both layers and broilers, and alter bacterial metabolism in poultry [9, 10]. Among different types of probiotics, yeast, especially *Saccharomyces cerevisiae*, is most common in poultry feed. *S. cerevisiae* influences intestinal microflora and pathogenic inhibition, immunomodulation, intestinal histological changes, and some hematobiochemical parameters, thereby improving broiler growth performance [11].

On the other hand, plant extracts have recently gained popularity as animal feed due to the restriction of most AGPs [12]. Maximum plant extracts produce subordinate metabolites such as essential oils, tannins, saponins, and phenolic compounds [13]. Most herbal plants are both safe and affordable. Herbs commonly used include garlic, cloves, neem leaves, moringa leaves, sophora flavescens, ginger, peppermint, sage, mustard, fenugreek, etc. Among them, one of the plants that can be used as an alternative to antibiotic feed is *Moringa oleifera*. Not only is it a very good source of amino acids and vitamins, but it also has a variety of medicinal properties, including growth promotion and antimicrobial activity [14]. Moringa leaf, according to Moreno Mendoza et al. [15], has potential prebiotic effects as well as antioxidant phytochemicals such as chlorogenic acid and caffeic acid. It is a beneficial source of antioxidant compounds such as ascorbic acid, flavonoids, carotenoids, and phenolics and is widely available in many tropical countries [16]. Moringa leaves contained 27.52% crude protein, 19.26% crude fiber, 2.24% crude fat, 7.12% ash, 76.52% moisture, and 43.88% carbohydrates. The iron and calcium levels were 28.29 and 20.09 mg/100g (DM), respectively [17]. *Moringa oleifera* leaf meal inclusion in broiler diets significantly increased the growth performance, carcass weight, and dressing percentage of broilers [18].

Therefore, growing concerns over antimicrobial resistance, along with restrictions on the use of antibiotic growth promoters, have encouraged the exploration of natural feed additives such as moringa and yeast as viable alternatives in poultry production systems to produce safe meat.

Although numerous studies have examined the effects of antibiotics, probiotics, and medicinal plants (*M. oleifera*) on broilers individually, no comparative evaluation of these three approaches on broiler growth performance, carcass traits, and meat quality has yet been conducted. Accordingly, this study was designed to test the hypothesis that a comparative assessment would help identify the most effective substitutes for antibiotics in broiler production.

## 2. Materials and Methods

### 2.1. Ethical approval and experimental materials

The present study was conducted at the Shahjalal Animal Nutrition Field Laboratory, Bangladesh Agricultural University, Mymensingh-2202, Bangladesh. Experimental procedures and bird handling were approved by the Institutional Animal Ethics Committee (approval no.: AWEEC/BAU/2025(2)/62(a)). Antibiotic (neomycin sulfate) used in the trial were obtained from a certified pharmaceutical supplier in Mymensingh Sadar (trade name: Neoxel Vet, company name: Sk + F). Fresh Moringa (*M. oleifera*) leaves were collected from the campus of Bangladesh Agricultural University, then sun-dried appropriately. Active dried baker’s yeast (*S. cerevisiae*) was procured from Angel Yeast Co., Ltd., China (*S. cerevisiae* E491, 1 × 10^10^ CFU/gm).

### 2.2. Preparation of broiler diet

The least-cost ration was formulated with commonly available ingredients throughout the experimental period. The ingredients used and the calculated nutrient composition of the broiler diet are shown in Table 1 below.

**Table 1.** The ingredients used and the nutrient composition of the broiler ration.

Ingredients	Amount (kg)	Calculated Chemical Composition	Amount (%)
Corn	48	CP	21.98
Soybean Meal	39.5	^*^ME (kcal/kg)	3030.55
Rice Polish	4	CF	5.16
Oil	5	▪Ca%	0.77
Limestone	1	▪Available P%	0.679
Dicalcium Phosphate	1.5	Methionine	0.53
Vitamins Mineral premixes	0.3	Lysine	1.23
Methionine	0.07		
Lysine	0.2		
Choline Chloride	0.15		
Mycotoxin Binder	0.03		
Salt	0.25		
**Total**	**100**		

*Calculated using the formula of Wiseman (1987); *▪* According to NRC [19] ^a^ 2.5 gm vitamin-mineral premix: Vitamin A, 13,500 I.U.; Vitamin D3, 1,500 I.U.; α-DL-tocopherol acetate, 50 mg; Menadione, 2 mg; Thiamine, 3 mg; Riboflavin, 5 mg; Pyridoxine, 4 mg; Cobalamin, 15 µg; Folic acid, 200 µg; Nicotinic acid, 60 mg; Ca-pantothenate, 30 mg; Choline, 750 mg; Ascorbic acid, 150 mg.

### 2.3. Experimental birds and other management practices of the birds

The trial on feed was conducted for 35 days with 240 (Indian River) day-old broiler chicks. Chicks were obtained from Kazi Farms Group. The average weight of the DOC was 38 gm. For the first seven days of the experiment, chicks were supplied with pre-starter crumble feed containing 23% CP and 2950 kcal ME/kg of diet for adaptation. After 7 days, a hand-mixed ration was prepared and supplied to the birds from day 8 to 35 (Table 1). The formulated hand-mixed diet was prepared according to NRC [19]. At the end of the adaptation period, healthy chicks were selected, weighed, and divided into different dietary treatment groups. There were 4 dietary treatment groups (CD, CDM, CDY, and CDA), each consisting of 60 chicks, with six replicates per treatment (10 birds/replicate). The dietary treatments were formulated as follows.

CD: Control Diet (Hand-mixed ration)

CDM: Control diet + 2.5% moringa leaf powder

CDY: Control diet + 2.5% yeast

CDA: Control diet + 0.015% antibiotic

The 2.5% inclusion (Yeast and Moringa) level was selected based on previous studies reporting optimal growth performance and gut health improvement within the 1–3% supplementation range [20, 21, 22]. The antibiotic neomycin sulfate was selected as the antibiotic growth promoter due to its broad-spectrum antibacterial activity and its approved veterinary use and common field application in local poultry production systems. Dietary supplementation with moringa, yeast, and antibiotics was maintained throughout the entire experimental period.

Birds were housed in a hygienic, naturally ventilated building with concrete walls. A total of 24 cages were used for the experiment, each providing a floor space of 0.91 m^2^ (120 cm × 76 cm). Prior to the arrival of chicks, the poultry house floors and cages were thoroughly cleaned and washed using pressurized water through a hosepipe, followed by disinfection with bleaching powder. All feeders, drinkers, containers, and other essential equipment were also properly cleaned, disinfected with phenyl solution, air-dried, and left unused for one week before chick placement. Sawdust was used as litter material and spread over the concrete floor to a depth of 5 cm. The litter was stirred weekly to prevent the accumulation of ammonia gas. A footbath was maintained at the entrance of both the poultry compound and the house to ensure biosecurity. An electric bulb was used for brooding the chicks. Birds were vaccinated against Newcastle disease and Gumboro disease according to the recommended vaccination schedule. Throughout the experimental period, birds were provided ad libitum access to fresh water and feed. Basal starter diets (Table 1) were formulated to meet the birds’ nutritional requirements.

### 2.4. Chemical Analysis

Representative samples of the experimental ration of each treatment were subjected to proximate analysis to determine proximate components, namely dry matter (DM), crude protein (CP), crude fiber (CF), ether extract (EE), nitrogen-free extract (NFE), & ash content according to the method of [23].

### 2.5. The experimental procedure and data collection

#### 2.5.1. Proximate Analysis

The proximate components and nutrient composition of the basal diets were determined according to the methods described by [23]. All samples were analyzed in triplicate, and the results were expressed as percentages. Crude protein content was determined using the Micro-Kjeldahl method [24].

#### 2.5.2. Growth performance

The preliminary body weights of the chicks were recorded at the commencement of the study using a digital weighing balance with appropriate precision (Model: HESA 3303) and subsequently measured every week to calculate body weight gain (BWG). Feed intake was determined by subtracting the amount of leftover feed from the amount offered in the previous morning. The feed conversion ratio (FCR) was calculated as the total feed consumed divided by the corresponding weight gain measured at the end of the starter, finisher, and overall production phases.

#### 2.5.3. Carcass evaluation

After completion of the feeding trial, one bird per replication was humanely slaughtered for carcass evaluation. To ensure empty gastrointestinal tracts, feed and drinking water were withdrawn overnight. The weights of the carcass components were then recorded.

#### 2.5.4. Meat quality

The meat quality of birds was analyzed in the laboratory of the Animal Science Department, Bangladesh Agricultural University. At first, meat samples were placed in a plastic bag, sealed, chilled in a refrigerator, and then stored at 4 °C for 24 h. The meat superiority characters, like the pH, water-holding capacity, cooking loss, drip loss, and thiobarbituric acid reactive substance (TBARS) assay, were evaluated.

#### 2.5.5. pH measurement

About 30 gm samples were first blended and then diluted with the required amount (200 ml) of distilled water, and then the pH of the meat was measured using a calibrated digital pH meter (HANNA H1 2211 pH/ORP).

#### 2.5.6. Determination of breast and thigh meat color

The carcasses were refrigerated for 24 h, and the color of each breast meat sample was measured individually using a Konica Minolta Chroma Meter CR-410 (Konica Minolta, Inc., Tokyo, Japan).

#### 2.5.7. Cooking loss

The samples were weighed and sealed in a double-layer polythene bag. Then kept in a water bath at 80 °C for 30 min. After 30 min, samples were removed from the water bath and spread for 10 min to allow surface drying. Then, the samples were weighed again. Then, the cooking loss was measured by using the following formula:


\[
{\rm Cooking\, Loss}\left({\%}\right) = \frac{{{\rm Sample\, weight\, before\, cooking}} - {\rm Sample\, weight\, after\, cooking}}{{{\rm Sample\, weight\, before\, cooking}}} \times 100
\]

#### 2.5.8. Drip loss

Approximately 25 gm (wet weight) of uniformly shaped muscle was excised from the same location of the breast for each sample and immediately weighed to obtain the initial weight. Each sample was then suspended on a string and placed in an airtight container, followed by storage at 4 °C. After 24 h, the samples were retrieved and reweighed using a digital balance to determine the final weight. Drip loss was subsequently calculated using the following formula:


\[
{\rm Drip\, Loss}\left({\%} \right) = \frac{{{\rm Initial\, weight\, of\, the\, sample}} - {\rm final\, weight\, of\, the\, sample}}{\rm Initial\, weight\, of\, the\, sample} \times 100
\]

#### 2.5.9. Water-holding capacity (%)

The water-holding capacity (WHC) of the breast muscle was determined using a centrifugation assay [25]. Approximately 1 gm of breast muscle was cut into cubes from each replicate and placed into a centrifuge tube. The samples were then centrifuged at 3,000 × *g* at 4 °C for 10 min. WHC was calculated based on the amount of exuded water using the following formula:


\[
{\rm WHC}\left({\%}\right) = \frac{{{\rm Sample\, weight\, after\, centrifugation}}}{{{\rm Sample\, weight\, before\, centrifugation}}} \times 100
\]

#### 2.5.10. Thiobarbituric acid (TBARS) determination

The thiobarbituric acid value (TBA) was determined as per the extraction method described by Witte et al. [26].

#### 2.5.11. Meat composition

The nutritional composition of meat was measured according to the method described by AOAC [23] & Kohn and Allen [24]. Equal portions of breast and thigh meat were homogenized together, and the composite sample was analyzed for meat proximate composition.

#### 2.5.12. Nutrient digestibility

Acid-insoluble ash (AIA) was used as a marker to calculate the nutrient digestibility of the experimental diet. When comparative digestion trials were performed, the birds were fed twice daily. A common diet (Table 1) was supplemented with 1% acid-insoluble ash to determine the digestibility of the nutrients. Each diet was fed to broiler chicks from 31 to 35 days of age. After a preliminary period of three days, all the feces were collected daily at the time of feeding. The feces were dried in a forced-air oven at 60 °C for 48 h and ground before analysis. The chemical compositions of the diets are also analyzed in the laboratory. The techniques used for AIA determination were coded as VO. The technique described by Vogtmann et al. [27] (VO) has been successfully employed to estimate nutrient digestibility in non-ruminant animals.

#### 2.5.13. Tibia ash and bone mineralization

Only the right tibia was measured for weight and length, and the left tibia was used for mineral density, content, and ash. The shank length and diameter were measured by using slide calipers. Tibias were removed between the tibial-tarsal joint and the tibial-femoral joint. The tibia and shank were then autoclaved at 121 °C for 15 min, and any remaining flesh and cartilage caps were removed carefully by hand. The length and diameter at the center of the diaphysis were determined using calipers and then oven-dried at 105 °C for 24 h. The bones were then ignited at 650 °C for 13 h, and the percentage ash was calculated by using the following formula:


\[
{\rm \% Ash} = \frac{{{\rm The\, ash\, weight}}}{{\rm dried\, bone\, weight}}
\]

#### 2.5.14. Cost-benefit analysis

Cost-benefit analysis was performed by calculating the consideration of the expenses of chicks, feed ingredients, moringa leaves, yeast, litter, electric bulbs, disinfectant, other equipment, vaccines, etc. In this calculation, other costs such as labor, electricity, medicine, etc. were not included.

### 2.6. Statistical analysis

Raw experimental data were recorded using Microsoft Excel. All recorded and calculated data were subsequently analyzed using IBM SPSS Statistics 22. A one-way analysis of variance (ANOVA) based on a completely randomized design (CRD) was conducted to evaluate the significance of treatment effects. Mean comparisons among treatments were performed using Duncan’s Multiple Range Test (DMRT). Statistical significance was set at *p* < 0.05.

## 3. Results

### 3.1. Growth Performance

The effect of medicinal plants, probiotics, and antibiotics on body weight, feed intake, and feed conversion ratio is presented in Table 2. Final body weight, body weight gain, and average daily gain were significantly higher (*p* < 0.05) in the antibiotic-added group compared to the control, moringa, and yeast-supplemented groups. There was no significant difference found in the case of feed intake among all the treatment groups, but intake was comparatively lower in the CDA group. Additionally, feed conversion ratio was lower in the CDA group compared to CD, CDM, and CDY groups (*p* < 0.05).

**Table 2.** Influence of medicinal plants, probiotics, and antibiotics on the growth performance of broilers.

Parameters	CD	CDM	CDY	CDA	SEM	*p*-value
FBW (gm)	1558^b^ ± 16.2	1642^ab^ ± 61.5	1626^b^ ± 33.4	1736^a^ ± 85	23.56	0.028
BWG (gm)	1382^b^ ± 19.1	1458^ab^ ± 60.9	1440^ab^ ± 38.03	1549^a^ ± 85	22.83	0.040
ADG (gm)	49^b^ ± 0.68	52^ab^ ± 2.18	51^ab^ ± 1.36	55^a^ ± 3.06	0.81	0.040
FI (gm)	3124 ± 106	3152 ± 77.58	3071 ± 116	2931 ± 146	38.12	0.165
FCR	2.26^a^ ± 0.09	2.16^a^ ± 0.12	2.13^ab^ ± 0.06	1.89^b^ ± 0.05	0.043	0.001

Here, CD = control diet, CDM = control diet + 2.5% moringa leaf powder, CDY = control diet + 2.5% yeast, and CDA = control diet + 0.015% antibiotic. gm = gram; FBW = final body weight; BWG = body weight gain; ADG = average daily gain; FI = feed intake; FCR = feed conversion ratio; SEM = standard error of the mean; ^a, b, c^, means bearing uncommon superscripts in a row differ significantly.

### 3.2. Carcass characteristics

The impact of medicinal plants, probiotics, and antibiotics on the carcass characteristics of broilers is presented in Table 3. Here, the live weight of birds was the highest in the CDA group, but dressing yield, head weight, and neck weight were higher in the CDM and CDY groups (*p* < 0.05). Liver and drumstick weights were significantly higher in the CDA group (*p* < 0.05). But Shank, gizzard, heart, and spleen weights were not statistically significant (*p* > 0.05) for all treatment groups.

**Table 3.** Influence of medicinal plants, probiotics, and antibiotics on carcass characteristics of broilers.

Parameters (gm)	CD	CDM	CDY	CDA	SEM	*p*-value
Live weight	1523^c^ ± 47.52	1670^ab^ ± 52.67	1628^b^ ± 55.07	1760^a^ ± 58.18	28.88	0.004
Dressing yield (%)	65.09^b^ ± 0.86	69.60^a^ ± 0.15	69.17^a^ ± 1.5	68.77^a^ ± 2.38	0.87	0.009
Head	33.00^b^ ± 3.61	39.67^a^ ± 2.08	38.00^ab^ ± 2.00	35.67^ab^ ± 3.05	1.02	0.048
Neck	39.00^b^ ± 5.29	53.33^a^ ± 4.04	48.33^a^ ± 9.45	43.00^ab^ ± 8.84	3.16	0.020
Shank	34.00 ± 3.00	31.00 ± 2.64	33.67 ± 2.08	37.33 ± 5.77	1.13	0.285
Liver	34.00^c^ ± 1.73	38.67^bc^ ± 4.50	43.33^ab^ ± 3.21	47.33^a^ ± 2.88	1.70	0.005
Gizzard	52.00 ± 9.64	51.67 ± 6.02	56.33 ± 3.51	46.67 ± 2.51	1.82	0.349
Heart	9.33 ± 1.15	8.33 ± 0.57	12.00 ± 4.00	9.67 ± 2.08	0.71	0.336
Breast	135.33^b^ ± 4.93	198.67^a^ ± 26.15	181.67^a^ ± 13.65	192^a^ ± 25.24	8.88	0.016
Thigh	67.00^c^ ± 7.00	84.00^a^ ± 2.64	72.00^bc^ ± 5.56	80.00^ab^ ± 4.35	2.37	0.014
Spleen	1.00 ± 0.00	1.33 ± 0.57	1.67 ± 0.57	1.33 ± 0.49	0.14	0.487
Drumstick	69.00^b^ ± 2.64	73.67^b^ ± 4.04	72.00^b^ ± 8.18	85.33^a^ ± 7.63	2.34	0.048

Here, CD = control diet, CDM = control diet + 2.5% moringa leaf powder, CDY = control diet + 2.5% yeast, CDA = control diet + 0.015% antibiotic, SEM = standard error of the mean; ** = highly significant at 1% level (*p* < 0.01); ^a, b, c,^ means bearing uncommon superscripts in a row, differ significantly.

### 3.3. Meat quality

The effect of medicinal plants, probiotics, and antibiotics on the meat quality of broilers is given in Table 4. Cook loss was 26.81% and drip loss was 3.71% in the CDA group, which were significantly higher compared to the other experimental groups (*p* < 0.05). But water-holding capacity was highest in the case of the CDM group, followed by CDY, CD, and CDA groups, respectively. The pH and the TBARS values were significantly higher in the CDA group compared to the CDM and CDY groups, respectively (*p* < 0.05).

**Table 4.** Influence of medicinal plants, probiotics, and antibiotics on the meat quality of broilers.

Parameter	CD	CDM	CDY	CDA	SEM	*p*-value
Cooking loss (%)	25.62^a^ ± 0.46	22.57^b^ ± 1.42	24.47^b^ ± 0.95	26.81^a^ ± 0.25	0.52	0.002
Drip loss (%)	3.67^a^ ± 0.04	3.15^b^ ± 0.15	3.39^b^ ± 0.21	3.71^a^ ± 0.06	0.08	0.003
WHC (%)	92.37^b^ ± 0.57	94.95^a^ ± 0.69	93.63^ab^ ± 0.97	92.32^b^ ± 1.90	0.43	0.049
pH value	6.02^b^ ± 0.07	6.13^a^ ± 0.02	6.05^ab^ ± 0.07	6.00^b^ ± 0.04	0.02	0.048
TBARS Value	0.100^d^ ± 0.00	0.104^c^ ± 0.00	0.111^b^ ± 0.001	0.117^a^ ± 0.001	0.001	0.0001

Here, CD = control diet, CDM = control diet + 2.5% moringa leaf powder, CDY = control diet + 2.5% yeast, CDA = control diet + 0.015% Antibiotic, SEM = standard error of the mean; WHC = water holding capacity; TBARS = thiobarbituric acid reactive substance; ^a, b, c,^ means bearing uncommon superscripts in a row, differ significantly.

### 3.4. Meat color

Table 5 shows the influence of medicinal plants, probiotics, and antibiotics on the meat color of broilers. In breast meat, lightness was the highest in the CDY group compared to the control, moringa, and antibiotic groups (*p* > 0.05). The redness of breast meat was highly significant in the CDM group (*p* < 0.001), followed by the CDY, CD, and CDA groups, respectively.

**Table 5.** Influence of medicinal plants, probiotics, and antibiotics on the meat color of broilers.

Parameters	CD	CDM	CDY	CDA	SEM	*p*-value
Breast Meat Color						
L*	47.69 ± 1.16	47.45 ± 0.05	51.12 ± 6.56	46.82 ± 3.37	1.04	0.525
a*	5.41^b^ ± 0.1.20	7.17^a^ ± 0.88	5.97^b^ ± 0.51	4.90^b^ ± 0.95	0.55	0.002
b*	8.01 ± 020	8.43 ± 0.99	8.51 ± 2.75	7.30 ± 1.52	0.43	0.800
Thigh Meat Color						
L*	47.35 ± 5.50	54.33 ± 2.50	54.43 ± 4.20	46.45 ± 7.14	1.69	0.173
a*	4.68^b^ ± 1.05	7.71^a^ ± 0.09	4.83^b^ ± 0.12	4.28^b^ ± 0.47	0.44	0.0001
b*	8.51 ± 0.23	8.69 ± 0.22	8.65 ± 1.07	7.33 ± 0.91	0.35	0.528

Here, CD = control diet, CDM = control diet + 2.5% moringa leaf powder, CDY = control diet + 2.5% yeast, CDA = control diet + 0.015% antibiotic, SEM = standard error of the mean; L* = Lightness; a* = Redness; b* = Yellowness; ^a, b, c,^ means bearing uncommon superscripts in a row differ significantly.

In case of thigh meat color, the lightness was higher in the CDY group (*p* > 0.05), whereas redness was higher in the CDM group (*p* < 0.05) compared to the control and antibiotic groups. The yellowness of meat was 8.69 in the CDM group, which was higher compared to other experimental groups (*p* > 0.05).

### 3.5. Meat composition

**Figure 1.**
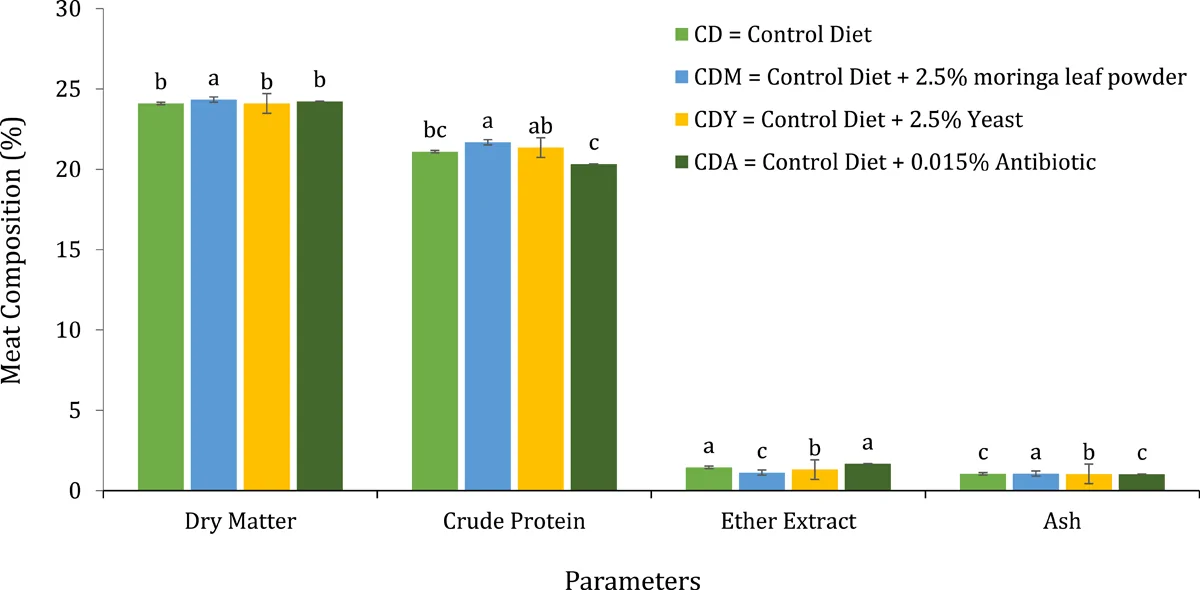
Influence of medicinal plants, probiotics, and antibiotics on the meat composition of broilers.

The nutrient composition of broiler meat is presented in Figure 1. DM, CP, and ash content were higher in the CDM group (24.34%, 21.68%, and 1.06%, respectively) compared to other groups. The percent of EE was lower in the CDM (1.12%) group and higher in the CDA (1.67%) group.

### 3.6. Nutrient digestibility

**Figure 2.**
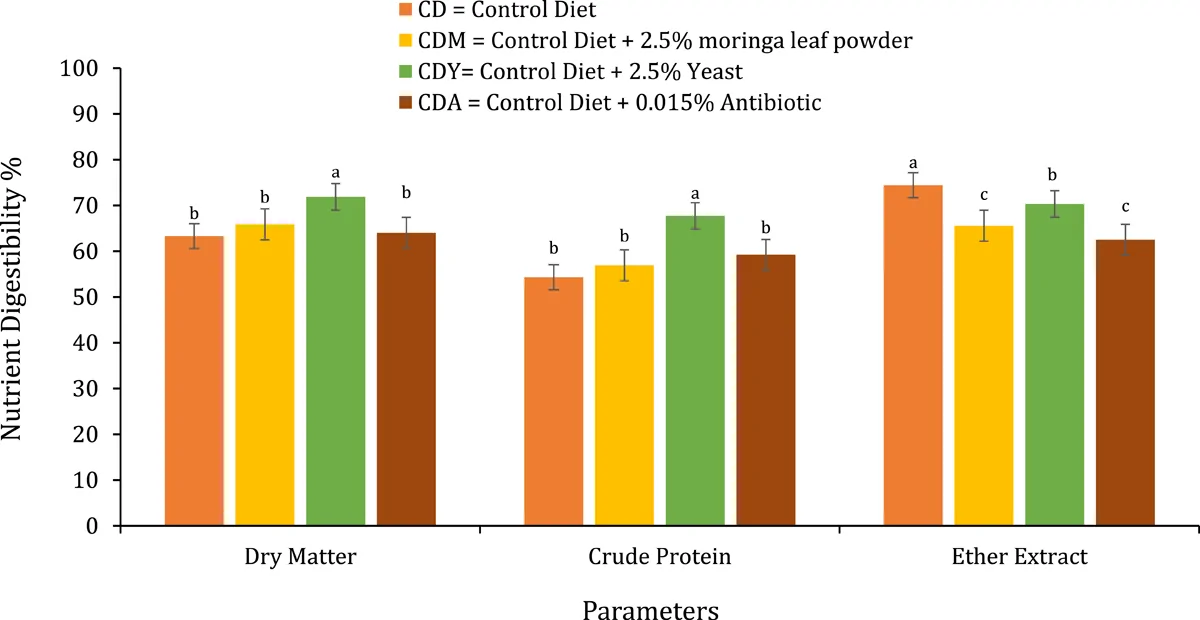
Influence of medicinal plants, probiotics, and antibiotics on the apparent nutrient digestibility of broilers.

The effect of medicinal plants, probiotics, and antibiotics on the apparent nutrient digestibility of broilers is presented in Figure 2. The DM, CP, and EE digestibility were significantly different in all dietary treatment groups. DM and CP digestibility were higher in the CDY group (71.86% and 67.71%, respectively). Ether extract digestibility was significantly higher in the CD group (*p* < 0.05) compared to the other dietary treatment groups.

### 3.7. Tibia ash and bone mineralization

Data are presented in Table 6. The length and width of the tibia in the CDA group were 9.58 cm & 1.04 cm, respectively, which were higher than those of the CDM, CD, and CDY groups (*p* < 0.05). Tibia weight was also higher in the CDA group (*p* < 0.05). However, the tibia ash percentage was significantly higher in the CDM group and subsequently in the CDY group compared to the CD and CDA groups (*p* < 0.05).

**Table 6.** Influence of medicinal plant, probiotic, and antibiotic on the tibia ash and bone mineralization of broilers.

Items	CD	CDM	CDY	CDA	SEM	*p*-value
Length (cm)	8.7^b^ ± 0.08	9.07^ab^ ± 0.15	8.53^b^ ± 0.37	9.58^a^ ± 0.32	0.218	0.002
Width (cm)	0.76^b^ ± 0.2	0.77^b^ ± 0.02	0.86^b^ ± 0.02	1.04^a^ ± 0.07	0.035	0.006
Weight (gm)	8.71^b^ ± 0.02	7.64^c^ ± 0.30	8.22^bc^ ± 0.21	11.58^a^ ± 0.36	0.252	0.003
ASH (%)	13.62^d^ ± 0.48	20.23^a^ ± 0.45	17.81^b^ ± 0.59	15.89^c^ ± 0.22	0.399	0.001

Here, CD = control diet, CDM = control diet + 2.5% moringa leaf powder, CDY = control diet + 2.5% yeast, CDA = control diet + 0.015% antibiotic, SEM = standard error of the mean; ^a, b, c,^ means bearing uncommon superscripts in a row differ significantly.

### 3.8. Cost-benefit ratio

Economic performance data of different dietary treatments are summarized in Table 7. Feed cost and total production cost were significantly lower (*p* < 0.00) in the CDA group and higher in the CD group compared to the moringa- and yeast-supplemented diets. The profit was significantly higher in the CDA group (0.30$) than CDY (0.18$), CDM (0.17$), and CD (0.12$).

**Table 7.** Cost-benefit ratio of broilers in different dietary treatments.

Parameters (USD$)	CD	CDM	CDY	CDA	SEM	*p*-value
Feed cost/kg LW	1.09 ± 0.02^a^	1.04 ± 0.03^ab^	1.03 ± 0.03^b^	0.91 ± 0.01^c^	2.56	0.00
Total production cost/kg LW	1.39 ± 0.01^a^	1.34 ± 0.02^ab^	1.33 ± 0.02^b^	1.21 ± 0.02^c^	2.56	0.00
Profit/kg	0.12 ± 0.02^c^	0.17 ± 0.01^bc^	0.18 ± 0.02^b^	0.30 ± 0.02^a^	2.56	0.00

Here, CD = control diet, CDM = control diet + 2.5% moringa leaf powder, CDY = control diet + 2.5% yeast, and CDA = control diet + 0.015% antibiotic. Sales price: TK. 185/kg live bird; 1 Taka = 0.0082 USD.

## 4. Discussion

The broilers used in this study were strictly maintained for research purposes and were not intended for human consumption and commercial marketing; therefore, an antibiotic withdrawal period was not applied. Similarly, moringa and yeast were also not withdrawn from the treatment groups. The antibiotic-supplemented feed yielded the greatest improvement in growth performance, whereas supplementation with moringa and yeast also resulted in significantly better outcomes compared to the control group. Although AGP supplementation resulted in superior growth performance, its use poses a risk to human health, as the continuous application of antibiotics accelerates the development of resistance in pathogenic bacteria affecting humans [28]. But medicinal herbs like moringa and prebiotics (such as yeast) don’t have any harmful effect on human health. The supplementation of moringa and yeast at 2–6% in feed for native chickens from 2–10 weeks of age can raise growth performance and feed efficiency and, conversely, decrease the number of pathogenic microorganisms in the intestines of birds [29]. Yeast improves the efficiency of the immune system, increases intestinal lumen health, and also improves digestion and absorption of nutrients, which results in better growth performance of broilers [30]. Hence, yeast can be used as a performance enhancer in broiler chickens instead of antibiotics.

The percent of dressing yield, the proportion of thigh, drumstick, and breast meat, was statistically high in moringa, followed by the antibiotic and yeast-supplemented group, & the lowest value was in the control group. These findings were similar to Abd El Latif et al. [31], who observed that different feed supplementation (moringa & yeast) has shown different carcass yields. Chicks‘ intake of dry yeast at a 0.3% level had a significantly higher effect on carcass weight as compared to all treatments [32].

Water-holding capacity, drip loss, and cooking loss are key indicators of meat quality, as essential nutrients can be lost through moisture exudation during storage and cooking [33]. Previous studies have demonstrated that dietary probiotics can enhance meat quality by improving the meat pH, tenderness, and color. Following slaughter, lactic acid accumulation in muscle tissue as a result of glycolysis causes a decline in pH, which is strongly associated with increased drip loss [34]. Because drip loss reflects the water-holding capacity of muscle, it represents an important determinant of overall meat quality [35]. In the present study, the antibiotic supplementation group appeared to reduce meat pH, while the addition of yeast and moringa-supplemented groups increased water-holding capacity and cooking yield by reducing drip loss and cooking loss, thereby enhancing meat quality compared to the antibiotic group. Dietary treatments may influence glycogen reserves and consequently the extent of pH decline. Lipid peroxidation is a major contributor to the deterioration of poultry meat quality, as it reduces nutritional value, impairs flavor and texture, and negatively affects the appearance of the meat [36]. MDA (malondialdehyde) is considered the main product of lipid peroxidation, which is also a measure of TBARS (thiobarbituric acid reactive substance) value by reactive oxygen species, the accumulation of which is usually used to monitor lipid peroxidation in poultry meat [37]. In this study, supplementation with moringa and yeast significantly decreased the TBARS values in thigh muscle, indicating a reduction in lipid peroxidation. This suggests that moringa and yeast supplementation may contribute to improved meat quality and extended shelf life. Similar results were also found by Ghasemi et al. [38], who showed that the value of TBARS in thigh meat after 30 days of storage at 4 °C was linearly decreased as the symbiotic inclusion concentrations in the diets increased. The declined MDA content may be related to the regulatory function of moringa and yeast on the fatty acid profiles of muscles. The higher TBARS value observed in the antibiotic group indicates increased lipid oxidation compared to the moringa and yeast-supplemented groups. This may be attributed to the absence of natural antioxidant compounds in the antibiotic diet. Moringa is rich in polyphenols, flavonoids, and vitamins with antioxidant properties, while yeast supplementation may enhance antioxidant enzyme activity and gut health, thereby reducing oxidative stress. In contrast, antibiotic supplementation does not provide antioxidative protection, which may explain the elevated lipid peroxidation in this group. Meat oxidation could reduce hydrolysis sensitivity, weaken protein degradation, and decrease water retention among myofibrils, which would subsequently increase the loss of juice from meat [39]. Therefore, the lower cooking loss of thigh recorded in this study may be associated with the simultaneously reduced MDA accumulation in the meat.

Meat color is an important sensory index because it affects consumers‘ first impression of meat [40]. High-quality meat has higher redness and lower lightness and yellowness [41]. In the present study, moringa and yeast additions increased the redness values but did not affect the lightness and yellowness values significantly. The slight increased yellowness observed in the moringa-supplemented group may be attributed to the high carotenoid content in *M. oleifera* leaves. However, Meng et al. [40] indicated that dietary probiotics could increase redness values but not lightness values, and Cheng et al. [42] reported that dietary synbiotics (including yeast cell wall, XOS, *Clostridium butyricum*, *B. licheniformis*, and *B. subtilis*) did not affect redness and lightness values. These differences may be related to the feeding stage, type, and level of treatment. However, further studies are needed to determine the exact reason for this finding.

The DM and ash content of meat samples in different treatments showed no significant differences, where CP content increased in the moringa-added group compared to the control and antibiotic-supplemented groups. The fat content was lower in the CDM group compared to the CDA group. In the same context, it was reported that dietary supplementation of MO silage improved the CP content and reduced the fat content of broilers [43]. This may be due to the capability of moringa leaf to improve protein absorption and retention [44]. The lower fat content in the CDM group is attributed to the difference in fat metabolism. Moringa supplementation decreases fat synthesis and prevents fat accumulation by inhibiting adipogenesis and helping in lipolysis [45]. This finding is consistent with studies by Hossain et al. [46] and Hossain et al. [47], who reported higher crude protein content in the breast meat of Alisma canaliculatum with probiotics (ACP 0.5%)-fed broilers. However, Hossain et al. [46] observed lower crude protein content in thigh meat of broilers that consumed a diet containing 0.5% and 1% ACP. In a recent report, Kim et al. [48] noted that ACP can increase the crude protein content of broiler breast meat.

The apparent digestibility of DM and CP was signiﬁcantly higher in yeast-supplemented groups. In contrast, digestibility improved in broilers fed moringa and yeast-supplemented feed compared to an antibiotic-supplemented diet. Yeast increases the density of beneficial caecal bacteria, such as *Ruminococcus* and *Bifidobacterium*, and reduces *Enterobacteriaceae*. So, supplementing broiler diets with 0.8% yeast culture (YC) optimized growth, feed efficiency, and nutrient digestibility and improved both feed efficiency and caecal microbiota diversity in broiler chickens [49]. This result is in conflict with the conclusion that supplementing broiler diets with *M. oleifera* leaf meal (MOLM) up to 25 gm/kg improved growth performance and conversion efficiency without affecting nutrient digestibility.

Although the length, width, and weight of the tibia were significantly higher in the antibiotic-added group, ash percentage was highly significant in the moringa and yeast-supplemented group. Prebiotics like yeast increase bone mineralization in broilers. Fermentation with yeast and additives improved nutrient profile, reduced fiber and phytate levels, and enhanced feed conversion ratio, which also exhibited greater tibia ash, indicating improved growth performance and bone health [50]. Dietary supplementation of *M. oleifera* leaf powder (12 gm/kg) increases the ash percentage of the tibia bone in broiler chickens [51].

The cost of production per kilogram of live bird was highest in the moringa-supplemented group; however, the antibiotic-supplemented group generated the greatest profit, exhibiting the highest cost–benefit ratio. Profit maximization remains a primary objective for poultry producers and can be achieved either by reducing input costs or increasing output to an optimal level. Consequently, if natural antioxidant additives are effective in producing healthier meat but do not provide sufficient economic returns, producers may be reluctant to incorporate herbal supplements into poultry feed. However, consumer preference has been shifting, and many consumers are now willing to pay a higher price for safe, antibiotic-free products, which may encourage producers to adopt herbal alternatives despite their higher production costs. The present investigation demonstrated that, when broilers were sold at market price, all treatment groups generated higher profitability than the control group. Furthermore, the supplemented groups offer additional advantages in terms of consumer health, as they provide a safer alternative to antibiotic use, and consumers are generally willing to pay a premium for safe, high-quality poultry products. Supplementation with natural antioxidants can help neutralize free radicals, enhance immune function, and increase micronutrient concentrations in tissues and bones.

Yeast supplementation has been shown to improve gut morphology and nutrient digestibility in broilers, likely through enhanced villus height and microbial balance, making nutrients more available for absorption compared with antibiotic treatments that mainly reduce pathogenic bacteria without directly enhancing digestive capacity [22]. *Moringa oleifera*, rich in antioxidants such as flavonoids and carotenoids, improves meat oxidative stability and color characteristics in poultry, but these quality improvements often do not translate into higher profitability due to increased feed costs and lack of premium pricing for quality traits in standard markets [20, 21].

## 5. Conclusions

The antibiotic group showed slightly higher profit due to better growth performance. However, the profit difference was relatively small and may not be economically substantial under practical conditions. Importantly, the moringa and yeast groups produced better meat quality and oxidative stability. Considering consumer health concerns and restrictions on antibiotic use, natural additives like moringa and yeast may be more sustainable and safer alternatives despite the slightly lower short-term profit. Therefore, moringa and yeast can be recommended for intensive rearing systems, particularly for farmers aiming to produce safe, high-quality broiler meat. Future research should further investigate their effects on disease resistance and performance across different broiler strains.

## Data Availability

The data presented in this study are available from the corresponding author upon reasonable request.

## Lists of abbreviations

AIA, acid insoluble ash; AGPs, antibiotic growth promoters; BWG, body weight gain; CF, crude fiber; CP, crude protein; Co., company; cm, centimeter; DM, dry matter; EU, European Union; EE, ether extract; FCR, feed conversion ratio; gm, gram; mg, milligram; kg, kilogram; kcal, kilocalorie; ME, metabolic energy; MDA, malondialdehyde; ml, milliliter; NFE, Nitrogen Free Extract; TBA, Thiobarbituric acid; min, minutes; TBARS, Thiobarbituric acid reactive substances; h, hour; I.U., International Units; L*, Lightness; a*, Redness; b* Yellowness; $, dollar; ºC, degree Celsius; *, 5% level of significance; **, 1% level of significance; *µ*g, Microgram.
